# Fluid Balance in Team Sport Athletes and the Effect of Hypohydration on Cognitive, Technical, and Physical Performance

**DOI:** 10.1007/s40279-017-0738-7

**Published:** 2017-05-15

**Authors:** Ryan P. Nuccio, Kelly A. Barnes, James M. Carter, Lindsay B. Baker

**Affiliations:** Gatorade Sports Science Institute, 617 W. Main St., Barrington, IL 60010 USA

## Abstract

Sweat losses in team sports can be significant due to repeated bursts of high-intensity activity, as well as the large body size of athletes, equipment and uniform requirements, and environmental heat stress often present during training and competition. In this paper we aimed to: (1) describe sweat losses and fluid balance changes reported in team sport athletes, (2) review the literature assessing the impact of hypohydration on cognitive, technical, and physical performance in sports-specific studies, (3) briefly review the potential mechanisms by which hypohydration may impact team sport performance, and (4) discuss considerations for future directions. Significant hypohydration (mean body mass loss (BML) >2%) has been reported most consistently in soccer. Although American Football, rugby, basketball, tennis, and ice hockey have reported high sweating rates, fluid balance disturbances have generally been mild (mean BML <2%), suggesting that drinking opportunities were sufficient for most athletes to offset significant fluid losses. The effect of hydration status on team sport performance has been studied mostly in soccer, basketball, cricket, and baseball, with mixed results. Hypohydration typically impaired performance at higher levels of BML (3–4%) and when the method of dehydration involved heat stress. Increased subjective ratings of fatigue and perceived exertion consistently accompanied hypohydration and could explain, in part, the performance impairments reported in some studies. More research is needed to develop valid, reliable, and sensitive sport-specific protocols and should be used in future studies to determine the effects of hypohydration and modifying factors (e.g., age, sex, athlete caliber) on team sport performance.

## Key Points

**Table:** 

Significant hypohydration (>2% body mass deficit) has been reported most consistently in soccer. Although other sports (e.g., American Football, rugby, basketball, tennis, and ice hockey) have reported high sweating rates, fluid balance disturbances have generally been mild, suggesting that drinking opportunities were sufficient to provide most athletes with enough fluid to offset significant fluid losses.
The effect of hydration status on team sport performance has been mixed. However, it seems that hypohydration is more likely to impair cognition, technical skill, and physical performance at higher levels of body mass loss (3–4% difference between trials) and when the method of dehydration involves heat stress.
Although exact mechanisms are unclear, increased subjective ratings of fatigue and perceived exertion consistently accompany hypohydration in team sport studies and could explain, in part, the performance impairments reported in some studies.

## Introduction

Body water is lost as a consequence of thermoregulatory sweating, and when fluid intake is insufficient to replace sweat losses, hypohydration (a body water deficit) occurs. Since evaporation of sweat is the primary avenue of heat loss during exercise, fluid losses and the risk of hypohydration in athletes can be significant. The rate of sweat loss is directly related to exercise intensity (metabolic heat production) [1]. Team sports, which are characterized by intermittent bursts of high-intensity exercise over prolonged periods of time (~1–2 h), can elicit heavy sweat losses [2, 3]. Other factors that are associated with increased sweating, such as large body mass [4, 5], hot/humid environments [1], and wearing protective clothing/equipment [6, 7], are also present in many team sports. Thus, it is not surprising that some of the highest sweating rates in athletes have been reported in team sports [3, 8]. However, individual sweating rates vary considerably [9, 10], as do the fluid intake habits of athletes and the in-game fluid replacement opportunities across sports [11]. Thus, the level of hypohydration incurred in team sport athletes can also vary substantially [10, 11]. Many studies have measured fluid balance in team sport athletes; however, few have presented a comprehensive summary of the literature [11]. It would be of interest to compare the levels of hypohydration incurred across sports to determine in which team sport(s) hydration education is potentially of greater concern.

It is well established that hypohydration (>2% body mass loss; BML) can impair endurance performance, particularly in hot/humid environments [9, 12]. However, the impact of hypohydration on an athlete’s performance during team sport competition is less clear. Performance in many team sports is dependent upon cognitive function (e.g., attention, decision making, memory, and reaction time), the execution of sport-specific technical skills (e.g., shooting, passing, and dribbling in soccer), and high-intensity physical abilities (e.g., sprinting, lateral movement, jumping, intermittent high-intensity running capacity). While studies have investigated the effect of hypohydration on some of these aspects of team sport performance, no papers have reviewed and discussed them collectively. There is a need to better understand the potential impact of hypohydration on sport-specific performance to help inform practical recommendations around fluid balance and team sports performance.

The aims of this paper are to: (1) provide a compilation of the fluid balance changes observed in team sport athletes during training and competition, (2) review the literature assessing the impact of hypohydration on cognitive, technical, and physical performance in team sports, (3) briefly discuss the potential mechanisms by which hypohydration could impact team sport performance, and (4) comment on current study limitations and considerations for future directions.

## Methodological Aspects

### Literature Search Criteria

To locate relevant articles for this review the literature search was conducted using PubMed and EBSCO databases. Multiple search phrases pertaining to “fluid balance”, “sweat losses”, “sweating rate”, “hypohydration”, “dehydration”, “team sport”, “performance”, “skill”, “cognition”, “attention”, “vigilance”, “decision making”, “memory”, “reaction time”, “intermittent”, “high-intensity”, “sprint”, “jump”, “power”, and “agility” were used. Other general inclusion criteria included English language and full-length articles published in peer-reviewed journals. Abstracts and unpublished observations were not included. The search period was through September 2016. A total of 75 original studies measuring sweating rate and/or fluid balance (involving ad libitum drinking, i.e., not controlled by study investigators) in athletes during training or competition were identified (see Table 1 for a general summary). The search located 20 original studies measuring the impact of hypohydration on performance during intermittent high-intensity protocols and involving team sport athletes as participants (see Tables 2 and 3 for details on individual studies).

**Table 1:** Summary of fluid balance studies in team sports

Risk for hypo-hydration^a^	Sport	Number of studies	References	Range in mean sweating rate (L/h)^b,c^	Range in mean fluid balance (% ∆ body mass)^b^	Duration of training/competition (h)^d^	Total number of athletes tested	Range in mean environmental conditions^b^	Range in mean age of athletes tested (years)^b^	Range in mean body mass of athletes tested (kg)^b^
High	Soccer	21	15–35	0.3–2.5	+0.4 to −3.5	1.1–2.5 T: *n* = 10 C: *n* = 8 T & C: *n* = 3	*n* = 497 (415 male, 82 female)	5–43 °C temp; 7–96% RH	10–27	37–80
	Rugby	7	67–73	0.4–2.0	−0.1 to −2.9	1.0–1.6 T: *n* = 1 C: *n* = 5 T & C: *n* = 1	*n* = 116 (male)	7–27 °C temp; 19–88% RH	20–28	76–107
Moderate	American Football	13	36–48	0.6–2.9	−0.1 to −2.4	2.0–4.5 T: *n* = 13	*n* = 225 (male)	22–35 °C temp; 43–92% RH	12–28	53–136
	Australian Rules Football	2	79, 80	0.9–2.1	−1.8 to −3.0	1.7 C: *n* = 2	*n* = 37 (male)	14–38 °C temp; 25–52% RH	NA	79–82
	Tennis	10	49–58	0.6–2.6	−0.2 to −1.4	1.1–2.0 T: *n* = 1 C: *n* = 9	*n* = 124 (98 male, 26 female)	17–37 °C temp; 32–62% RH	14–24	52–81
	Ice hockey	5	74–78	0.7–1.8	−0.8 to −1.3	1.0–2.2 T: *n* = 4 C: *n* = 1	*n* = 117 (male)	3–14 °C temp; 30–66% RH	17–21	80–90
	Field hockey	1	89	0.6	−0.2 to −0.5	1.9 C: *n* = 1	*n* = 16 (female)	22–23 °C temp; 51–57% RH	19	61
Low	Basketball	9	15, 59–66	0.7–2.7	−0.6 to −1.6	1.0–2.8 T: *n* = 4 C: *n* = 4 T & C: *n* = 1	*n* = 230 (189 male, 41 female)	17–30 °C temp; 20–60% RH	15–24	68–99
	Gaelic Football	1	86	1.4	−1.1	1.3 T: *n* = 1	*n* = 20 (male)	17 °C temp; 85% RH	~27	NA
	Cricket	3	81–83	0.1–1.4	+0.1 to −4.3	4.0 T: *n* = 1 C: *n* = 2	*n* = 68 (50 male, 18 female)	23–33 °C temp; 22–77% RH	20–22	68–87
	Baseball	2	84, 85	0.7–0.8	−1.3	2.0–3.8 T: *n* = 1 T & C: *n* = 1	*n* = 16 (male)	32–37 °C temp	21	61–64
	Beach volleyball	1	87	2.0	−0.8	0.7 C: *n* = 1	*n* = 47 (male)	34 °C temp; 56% RH	26	83
	Court volleyball	1	88	0.6	−0.4	2.0 T: *n* = 1	*n* = 36 (female)	NA	15	61
	Futsal	1	17	0.4–0.5	−0.4 to −0.5	1.2–1.3 T: *n* = 1	*n* = 26 (male)	31 °C temp; 53% RH	9–11	35–42
	Netball	1	15	0.7–1.0	−0.3 to −0.9	1.2–1.8 T & C: *n* = 1	*n* = 22 (female)	17–28 °C temp; 30–66% RH	~20	74
	Water polo	1	91	0.3–0.8	−0.3 to −0.4	NA T & C: *n* = 1	*n* = 23 (male)	24 °C air temp; 54–70% RH; 27 °C water temp	~24	88
	Badminton	1	90	1.0–1.1	−0.3 to −0.4	0.6–0.7 C: *n* = 1	*n* = 70 (46 male, 24 female)	24 °C temp; 50% RH	23	59–75

^a^Risk for hypohydration is based upon the criteria specified in Fig. 1

^b^The ranges include mean values reported from each study as well as sub-groups or separate training sessions/matches within studies, where applicable

^c^Where sweating rates were not reported they were estimated from total sweat loss and exercise duration provided in the original paper (in studies where total exercise duration was not reported, sweating rates were not calculated)

^d^The *n* represents the number of studies that were conducted during training (T) and competition (C)

*NA* not available, *RH* relative humidity, *temp* temperature

**Table 2:** Summary of studies measuring effects of hypohydration on cognitive performance and technical skill during team sports

Reference	Sport	Subjects	Protocol	Hydration levels (% ∆ body mass)	Physiological and subjective measures	Effect of hypohydration on sport-specific technical skill	Effect of hypohydration on cognition	Potential study limitations
Ali et al. 2011 [106]	Soccer	*n* = 10, 26 y female Premier division players	LIST protocol (90 min) with water intake (total of 15 ml/kg) or no fluid LSPT performed before, during, and after LIST protocol Env conds: NA	−2.2% (no fluid), −1.0% (water intake)	Blood lactate, HR, RPE, and Tc higher in no fluid trial NS: pleasure/displeasure (Feeling Scale), perceived activation (Felt Arousal Scale), thermal comfort	NS: Passing	NA	Subjects not blinded to hydration status No control (EUH) trial
Bandelow et al. 2010 [93]	Soccer	*n* = 20, 20 y male university players	Ad libitum water intake or encouraged to drink readily available water and sports drink during a match Battery of cognitive tests performed before, at halftime, and after match Env conds: 34 °C, 62–65% RH	Down to −2.5%	NA	NA	HYPO impaired working memory simple reaction time (Sternberg test) NS: Fine motor speed (finger tapping test), visuospatial working memory (Corsi block test), visuomotor reaction time (visual sensitivity test)	No control (EUH) trial Cognitive test not sport-specific
Edwards et al. 2007 [94]	Soccer	*n* = 11, 24 y male moderately active players	45 min cycling, then 45 min soccer match with water intake (80% replacement of fluid losses), water mouth rinse, or no fluid Mental concentration test performed after match Env conds: Cycling: 24–25 °C, 47–55% RH Match: 19–21 °C, 46–57% RH	−2.4% (no fluid), −2.1% (mouth rinse), −0.7% (water intake)	Tc higher during match in no fluid vs. water intake trial RPE higher during no fluid vs. water intake and mouth rinse trials	NA	NS: Mental concentration (number identification)	Subjects not blinded to hydration status Cycling exercise prior to match not realistic to soccer Cognitive test not sport-specific
McGregor et al. 1999 [95]	Soccer	*n* = 9, 20 y male semiprofessional players	LIST protocol (90 min) with fluid intake (total of 15 ml/kg, sugar-free lemon drink) or without fluid Soccer skill test and mental concentration test performed before and after LIST Env conds: 13–20 °C, 57% RH	−2.4% (no fluid), −1.4% (fluid intake)	HR and RPE higher during no fluid trial	5% decrease in dribbling skill performance (longer time to completion) with no fluid; maintenance of skill with fluid	NS: Mental concentration (number identification)	Subjects not blinded to hydration status No control (EUH) trial Cognitive test not sport-specific
Owen et al. 2013 [105]	Soccer	*n* = 13, 22 y male semiprofessional players	LIST protocol (90 min) with prescribed water intake (to replace 89% of sweat losses), ad libitum water intake, or no fluid LSST and LSPT performed before and after LIST protocol Env conds: 19.4 °C, 59.4% RH	−2.5% (no fluid), −1.1% (ad libitum water intake), −0.3% (prescribed water intake)	RPE higher during no fluid vs. prescribed water intake HR higher during no fluid vs. prescribed water intake and ad libitum water intake	NS: Passing and shooting skill	NA	Subjects not blinded to hydration status
D’Anci et al. 2009 [100]	Rowing, lacrosse, and American Football	Study 1: 16 male (20 y) and 15 female (21 y) college athletes Study 2: 12 male (20 y) and 12 female (19 y) college athletes	Water intake to maintain EUH or no fluid intake during a hard 60–75 min natural (coach-run) practice in 2 studies Cognitive test battery performed after practice	Study 1: −1.8% (HYPO), −0.1% (EUH) Study 2: −1.2% (HYPO), +0.1% (EUH)	Study 1: Thirst higher with HYPO; POMS vigor lower with HYPO; POMS anger, fatigue, depression, and tension higher with HYPO Study 2: Thirst higher with HYPO; POMS vigor lower with HYPO; POMS anger, depression, and tension higher with HYPO	NA	Vigilance (continuous performance test) impaired (by 3–4%) in HYPO trials of Study 1 only NS: Short-term memory (digit span task), spatial memory (map memory test), simple reaction time, choice reaction time, mathematical addition, visual perception (mental rotation task), and map planning of Study 1 and 2	Subjects not blinded to hydration status Practice sessions not standardized between trials Cognitive test not sport-specific
MacLeod and Sunderland 2012 [98]	Field hockey	*n* = 8, 22 y female players on a national or international team (elite)	Day 1: 2 h passive heat stress (39.9 °C, 73% RH) followed by controlled fluid intake to induce HYPO or EUH Day 2: 60-min intermittent treadmill protocol designed to mimic demands of field hockey (ad libitum water intake) Field hockey skill measured at baseline (day 1) and before and after treadmill protocol (day 2), skill test involved dribbling, passing, and shooting (at randomly illuminated target) Env conds: Treadmill protocol: 33.3 °C, 59% RH Skills in gym: 16.3–22.2 °C	~ −2% (HYPO trial), ~0% (EUH trial) at start of day 2 No difference in ad libitum water intake on day 2 (88% vs. 80% replacement of fluid losses)	RPE and thirst higher with HYPO before treadmill protocol NS: HR, Tc	NS: Field-hockey skill performance	Decision-making time during the skill test was 7% slower with HYPO vs. EUH before the treadmill protocol NS: Decision-making time after the treadmill protocol	Subjects not blinded to hydration status Intermittent protocol was on a treadmill and therefore not sport-specific Method of dehydration (passive heat stress previous day) may not be ecologically valid
Burke and Ekblom 1984 [112]	Tennis	*n* = 10, 5 male (21–32 y) and 5 female (30–40 y) healthy, active tennis players	Water (505 ml) or no fluid intake during 2-h simulated tennis matches Tennis skill test (accuracy of 50 shots hit off a ball machine) performed before and after match Env conds: Indoors, 23–25 °C	−2.7% (no fluid), −1.1% (water intake)	NA	NS: Tennis shot accuracy	NA	Subjects not blinded to hydration status No control (EUH) trial No validity or reliability testing of the tennis skill test
Baker et al. 2007 [97]	Basketball	*n* = 11, 17–28 y male competitive players	3-h interval walking in heat chamber (to establish 1–4% HYPO or maintain EUH) prior to 80-min simulated game Subjects drank water to maintain target HYPO level or flavored water in EUH trial TOVA at baseline, after heat chamber, and after game Env conds: Heat chamber: 40 °C, 20% RH Game: indoors, temperate	−1%, −2%, −3%, and −4% (HYPO trials), 0% (EUH trial)	More lightheaded, hot/overheated, and total body fatigue in HYPO trials (mean of 1, 2, 3, and 4%) NS: Tc	NA	More TOVA omission errors and commission errors and slower response time (by 6–8%) in HYPO trials (mean of 1, 2, 3, and 4%)	Subjects not blinded to hydration status Exercise-heat stress prior to TOVA not realistic to basketball Cognitive test not sport-specific
Baker et al. 2007 [102]	Basketball	*n* = 17, 17–28 y male competitive players	3-h interval walking in heat chamber (to establish 1–4% or maintain EUH) prior to 80-min simulated game Subjects drank water to maintain target HYPO level or flavored water in EUH trial Shooting drills completed throughout simulated game; drills included stationary shots (spot-up 3-point shots, 15-ft shots, and free throws) and shots on the move (off the dribble 15-ft jump shots and layups) Env conds: Heat chamber: 40 °C, 20% RH Game: indoors, temperate	−1%, −2%, −3%, and −4% (HYPO trials), 0% (EUH trial)	More leg fatigue and lightheaded in 3 and 4% HYPO trials vs. EUH trial More upper and total body fatigue in 4% HYPO trial vs. EUH trial Higher Tc during 2nd quarter of 4% HYPO trial vs. EUH trial NS: RPE and HR	Fewer shots on the move made in 3% (by ~9%) and 4% (by ~12%) HYPO trials vs. EUH trial Fewer stationary shots made in 4% HYPO trial vs. EUH trial For all shots combined significantly fewer shots made in 2% (by 7%), 3% (by 9%), and 4% (by 12%) HYPO trials vs. EUH trial Progressive decrease in total number of total shots made with increasing BML (from 2% to 4% HYPO) NS: Shooting percentage for shots on the move and stationary shots in 1, 2, 3, and 4% HYPO trials vs. EUH trial	NA	Subjects not blinded to hydration status Exercise-heat stress prior to simulated game not realistic to basketball No validity or reliability testing of simulated game/basketball drills
Brandenburg and Gaetz 2012 [63]	Basketball	*n* = 17, 24 y female national level players (elite)	Descriptive study: *Ad libitum* intake of water and/or sports drink (diluted per individual preference) during 2 international games Env conds: Indoors, 22.5–23.5 °C, 44–50% RH	−2.1 to +0.5% (game 1), −2.0 to +0.1% (game 2)	NA	Significant inverse relation between field goal percentage and BML in game 2 (*r* = −0.61) NS: Field goal percentage in game 1	NA	No control (EUH) trial Potential confounding effect of CHO on field goal percentage
Carvalho et al. 2011 [60]	Basketball	*n* = 12, 14–15 y male players on Portuguese national team	90-min training session with ad libitum water or no fluid Basketball drills performed before and after the training session Env conds: Indoors, 21.9–26.0 °C, 48.3–54.1% RH	−2.5% (no fluid), −1.1% (ad libitum water intake)	RPE higher in no fluid trial	NS: 2-pt, 3-pt, and free throw shooting percentage However, 2-pt field goal percentage 5.8% lower in no fluid vs. ad libitum water trial	NA	Subjects not blinded to hydration status No control (EUH) trial No validity or reliability testing of the basketball drills
Dougherty et al. 2006 [103]	Basketball	*n* = 15, 12–15 y male competitive players	2 h interval walking/cycling in heat chamber (to establish 2% HYPO or maintain EUH) prior to 60-min simulated game Subjects drank water to maintain 2% HYPO or flavored water in EUH trial Shooting drills (3-point shots, 15-ft shots, free throws, and layups) completed throughout simulated game Env conds: Heat chamber: 35 °C, 20% RH Game: indoors, temperate	−2% (HYPO trial), 0% (EUH trial)	More upper body fatigue and higher HR and Tc in HYPO trial vs. EUH trial NS: RPE and total body fatigue	Overall shooting percentage for long-range shots (3-point shots, 15-ft shots, and free throws) lower (by 8%) in HYPO trial NS: layup shooting percentage	NA	Subjects not blinded to hydration status Exercise-heat stress prior to simulated game not realistic to basketball No validity or reliability testing of simulated game/basketball drills
Hoffman et al. 1995 [101]	Basketball	*n* = 10, 17 y male players on Israel regional youth team	Water intake or no fluid during 2-on-2 full court games with controlled timing and controlled number of field goal and free throw attempts during games Env conds: Indoors, 20.8 °C, 64% RH	−1.9% (no fluid), NA (water intake permitted)	NS: RPE or HR	NS: Field goal and free throw percentage However, there was a 8.1% decrease in field goal % from 1st to 2nd half in HYPO trial and 1.6% increase in water intake trial (net difference of 9.7% between hydration states)	NA	Subjects not blinded to hydration status % ∆BM during water intake trial not reported; unclear whether water intake trial was ad libitum or a control (EUH) trial No validity or reliability testing of 2-on-2 basketball game
Hoffman et al. 2012 [96]	Basketball	*n* = 10, 21 y female division 1 college players	Water to replace fluid losses or no fluid intake during a 40-min live scrimmage Shooting circuit, lower body reactive agility (Quick Board), and visual reaction time (Dynavision D2) performed before and after the scrimmage Env conds: Indoors, 22.6 °C, 50.9% RH	−2.3% (no fluid), NA (water intake)	NS: HR and player load (Catapult GPS)	NS: Number of shots made	Impaired lower body reactive agility performance in no fluid vs. water intake trial NS: visual reaction time	Subjects not blinded to hydration status % ∆BM during water intake trial not reported; unclear whether EUH was maintained Cognitive tests not sport-specific (albeit still potentially relevant) No validity or reliability testing of the basketball shooting circuit or cognitive tests
Devlin et al. 2001 [109]	Cricket	*n* = 7, 21 y male, well-trained (medium-fast bowlers), skilled bowlers	Fluid restriction (30 ml flavored, colored ice blocks) or prescribed fluid intake (80% replacement of losses via flavored, colored water) during 1-h intermittent exercise Cricket bowling skill test before and after exercise Env conds: Exercise: 28 °C, 40% RH Skills test: 16 °C, 60% RH	−2.8% (HYPO trial), −0.5% (EUH trial)	RPE higher during HYPO trial at post-exercise bowl test NS: HR	Bowling accuracy, both bowling line (by 16.4%) and length of delivery (by 15.4%) impaired by HYPO NS: Bowling velocity	NA	Subjects not blinded to hydration status No validity or reliability testing of the cricket skill test
Gamage et al. 2016 [83]	Cricket	*n* = 30, 22 y male elite cricketers (8 batsmen, 10 fast-bowlers and 12 fielders)	Fluid restriction (4 ml/kg/h) or fluid provision (12–15 ml/kg/h) during 2 h of standardized cricket training Battery of cricket skills tests before and after training Env conds: Outdoors: 27.2–32.8 °C, 66–89% RH, ~2 mph wind speed	−3.7% (fluid restriction trial), −0.9% (fluid provision trial)	NA	Decrease in performance from pre- to post-training for bowling line speed (by 1.0%) and accuracy (by 19.8%), throwing speed (by 4.1%) and accuracy (22.3%) for sidearm technique, throwing speed (6.6%) and accuracy (14.2%) for overarm technique in the fluid restriction trials Performance was maintained (NS difference between pre- and post-training) in the fluid provision trials NS: bowling length	NA	Subjects not blinded to hydration status Type of fluid provided not reported No validity or reliability testing of the cricket skill test

Values are means or ranges where specified

*BM* body mass, *BML* body mass loss, *Env conds* environmental conditions, *EUH* euhydration, *GPS* global positioning system, *HR* heart rate, *HYPO* hypohydration, *LIST* Loughborough Intermittent Shuttle Test, *LSPT* Loughborough Soccer Passing Test, *LSST* Loughborough Soccer Shooting Test, *NA* not available, *NS* no significant effect, *POMS* Profile of Mood States, *RH* relative humidity, *RPE* rating of perceived exertion, *Tc* body core temperature, *TOVA* test of variables of attention

**Table 3:** Summary of studies measuring effects of hypohydration on physical performance (i.e., sprinting, jumping, lateral movements, and intermittent high-intensity running capacity) in team sports

Reference	Sport	Subjects	Protocol	Hydration levels (% ∆ body mass)	Physiological and subjective measures	Effect of hypohydration on physical performance	Potential study limitations
Ali et al. 2011 [106]	Soccer	*n* = 10, 26 y female Premier division players	LIST protocol (90 min) with water intake (total of 15 ml/kg) or no fluid 15-m sprints performed throughout LIST protocol Env conds: NA	−2.2% (no fluid), −1.0% (water intake)	Blood lactate, HR, RPE, and Tc higher in no fluid trial NS: pleasure/displeasure (Feeling Scale), perceived activation (Felt Arousal Scale)	NS: Mean sprint time	Subjects not blinded to hydration status No control (EUH) trial
Ali and Williams 2013 [113]	Soccer	*n* = 8, 24 y male university players	LIST protocol (90 min) with water intake (total of 15 ml/kg) or no fluid 15-m sprints performed throughout LIST protocol Env conds: NA	−3.7% (no fluid), −2.3% (water intake)	RPE higher in no fluid trial NS: HR and Tc; isometric strength, isokinetic strength, and muscle power of the knee flexors and knee extensors	NS: Mean sprint time	Subjects not blinded to hydration status No control (EUH) trial
Edwards et al. 2007 [94]	Soccer	*n* = 11, 24 y male moderately active players	45 min cycling, then 45 min soccer match with water intake (80% replacement of fluid losses), water mouth rinse, or no fluid Yo–Yo intermittent recovery test performed after match Env conds: Cycling: 24–25 °C, 47–55% RH Match: 19–21 °C, 46–57% RH	−2.4% (no fluid), −2.1% (mouth rinse), −0.7% (water intake)	Tc higher during match in no fluid vs. water intake trial RPE higher during no fluid vs. water intake and mouth rinse trials	Less distance covered during the Yo–Yo test in no fluid (by 13%) and mouth rinse trials (by 15%) vs. water intake trial	Subjects not blinded to hydration status Cycling exercise prior to match not realistic to soccer
McGregor et al. 1999 [95]	Soccer	*n* = 9, 20 y male semiprofessional players	LIST protocol (90 min) with fluid intake (total of 15 ml/kg, sugar-free lemon drink) or without fluid 15-m sprints performed throughout the LIST protocol Env conds: 13–20 °C, 57% RH	−2.4% (no fluid), −1.4% (fluid intake)	HR and RPE higher during no fluid trial	Mean sprint time was longer in the last 15-min block of the LIST protocol during the no fluid trial vs. fluid intake trial	Subjects not blinded to hydration status No control (EUH) trial
Owen et al. 2013 [105]	Soccer	*n* = 13, 22 y male semiprofessional players	LIST protocol (90 min) with prescribed water intake (to replace 89% of sweat losses), ad libitum water intake, or no fluid Yo–Yo intermittent recovery test performed before and after LIST protocol Env conds: 19.4 °C, 59.4% RH	−2.5% (no fluid), −1.1% (ad libitum water intake), −0.3% (prescribed water intake)	RPE higher during no fluid vs. prescribed water intake HR higher during no fluid vs. prescribed water intake and ad libitum water intake	NS: Distance covered during the Yo–Yo test	Subjects not blinded to hydration status
Burke and Ekblom 1984 [112]	Tennis	*n* = 10, 5 male (21–32 y) and 5 female (30–40 y) healthy, active tennis players	Water (505 ml) or no fluid intake during 2-h simulated tennis matches Sargent jump test (explosive power) performed before and after the matches Env conds: Indoors, 23–25 °C	−2.7% (no fluid), −1.1% (water intake)	NA	NS: maximum jump height and anaerobic power	Subjects not blinded to hydration status No control (EUH) trial
Baker et al. 2007 [102]	Basketball	*n* = 17, 17–28 y male competitive players	3 h interval walking in heat chamber (to establish 1–4% or maintain EUH) prior to 80-min simulated game Subjects drank water to maintain target HYPO level or flavored water in EUH trial Sprinting, lateral movement (defensive slides), and combination, and jumping drills completed throughout a simulated game Env conds: Heat chamber: 40 °C, 20% RH Game: indoors, temperate	−1%, −2%, −3%, and −4% (HYPO trials), 0% (EUH trial)	More leg fatigue and lightheaded in 3% and 4% HYPO trials vs. EUH trial More upper and total body fatigue in 4% HYPO trial vs. EUH trial Higher Tc during 2nd quarter of 4% HYPO trial vs. EUH trial NS: RPE and HR	Longer total sprint time in 2% (by ~7%), 3% (by ~8%), and 4% (by ~16%) HYPO trials vs. EUH Longer lateral movement time in 3% and 4% HYPO trials vs. EUH trial Longer combination drill time in 3% and 4% HYPO trials vs. EUH trial Longer repeated jump time in 4% HYPO trial vs. EUH trial NS: maximum vertical jump	Subjects not blinded to hydration status Exercise-heat stress prior to simulated game not realistic to basketball No validity or reliability testing of simulated game/basketball drills
Carvalho et al. 2011 [60]	Basketball	*n* = 12, 14–15 y male players on Portuguese national team	90-min training session with ad libitum water or no fluid Basketball drills performed before and after the training session Env conds: Indoors, 21.9–26.0 °C, 48.3–54.1% RH	−2.5% (no fluid), −1.1% (ad libitum water intake)	RPE higher in no fluid trial	NS: Sprinting and defensive slide times	Subjects not blinded to hydration status No control (EUH) trial No validity or reliability testing of the basketball drills
Dougherty et al. 2006 [103]	Basketball	*n* = 15, 12–15 y male competitive players	2-h interval walking/cycling in heat chamber (to establish 2% HYPO or maintain EUH) prior to 60-min simulated game Subjects drank water to maintain 2% HYPO or flavored water in EUH trial Sprinting, lateral movement (defensive slides), combination, and jumping drills completed throughout a simulated game Env conds: Heat chamber: 35 °C, 20% RH Game: indoors, temperate	−2% (HYPO trial), 0% (EUH trial)	More upper body fatigue and higher HR and Tc in HYPO trial vs. EUH trial NS: RPE and total body fatigue	Longer total and mean sprint times (by ~6%) and lateral movement times (by ~7%) in HYPO trial vs. EUH trial NS: combination drill time, maximum jump height, repeated jumping time	Subjects not blinded to hydration status Exercise-heat stress prior to simulated game not realistic to basketball No validity or reliability testing of simulated game/basketball drills
Hoffman et al. 1995 [101]	Basketball	*n* = 10, 17 y male players on Israel regional youth team	Water intake or no fluid during 2-on-2 full court games Squat jump, countermovement jump, and 30-sec anaerobic power jump test performed before, at halftime, and immediately after games Env conds: Indoors, 20.8 °C, 64% RH	−1.9% (no fluid), NA (water intake permitted)	NS: RPE or HR	NS: maximum vertical jump height during squat jump and countermovement jump, 30-s anaerobic power jump test However, anaerobic power during the 30-sec jump test was 19% lower in the no fluid vs. water intake trial after the game	Subjects not blinded to hydration status Unclear whether water intake trial was ad libitum or a control (EUH) trial No validity or reliability testing of 2-on-2 basketball game
Hoffman et al. 2012 [96]	Basketball	*n* = 10, 21 y female division 1 college players	Water to replace fluid losses or no fluid intake during a 40-min live scrimmage Countermovement jump performed before and after the scrimmage Env conds: Indoors, 22.6 °C, 50.9% RH	−2.3% (no fluid), NA (water intake)	NS: HR and player load (Catapult GPS)	NS: Peak and mean power during countermovement jump	Subjects not blinded to hydration status No validity or reliability testing of the reactive agility test
Devlin et al. 2001 [109]	Cricket	*n* = 7, 21 y male, well-trained, skilled bowlers	Fluid restriction (30 ml flavored, colored ice blocks) or prescribed fluid intake (80% replacement of losses via flavored, colored water) during 1 h intermittent exercise Maximal multi-stage shuttle run performed before and after exercise Env conds: Exercise: 28 °C, 40% RH Skills test: 16 °C, 60% RH	−2.8% (HYPO trial), −0.5% (EUH trial)	RPE higher during HYPO trial at post-exercise bowl test NS: HR	Fewer shuttles completed after intermittent exercise in HYPO trial vs. EUH trial (by 7.7%)	Subjects not blinded to hydration status No validity or reliability testing of the shuttle run
Gamage et al. 2016 [83]	Cricket	n = 30, 22 y male elite cricketers (8 batsmen, 10 fast-bowlers and 12 fielders)	Fluid restriction (4 ml/kg/h) or fluid provision (12–15 ml/kg/h) during 2 h of standardized cricket training Cricket batter running test (timed running between wickets) before and after training Env conds: Outdoors: 27.2–32.8 °C, 66–89% RH, ~2 mph wind speed	−3.7% (fluid restriction trial), −0.9% (fluid provision trial)	NA	Run time increased (slower performance) from pre- to post-training (by 2.2%) in the fluid restriction trial Run performance was maintained from pre- to post-training in the fluid provision trial	Subjects not blinded to hydration status Type of fluid provided not reported No validity or reliability testing of the cricket running test
Davis et al. 2015 [114]	Baseball	*n* = 8, 21 y male college players	Dehydration protocol (~90-min treadmill walking in the heat), followed by controlled rehydration to EUH or 3% HYPO. Intermittent sprinting (3 bouts of 8 × 30-m sprints with 30 s rest between sprints and 3 min between bouts) performance measured the morning after establishing EUH or HYPO Env conds: Treadmill walking: 38–39 °C, 30–40% RH Sprint performance: 22 °C, 25–32% RH	−3% (HYPO trial), 0% (EUH trial)	Higher HR and RPE in HYPO trial vs. EUH trial during bout 2 and 3 NS: HR and RPE during bout 1	Longer mean sprint time in HYPO trial vs. EUH trial during bout 2 (by ~3%) and bout 3 (by ~4%) NS: mean sprint speed during bout 1	Subjects not blinded to hydration status No measure of sprint performance at baseline (pre-HYPO) Dehydration protocol was on a treadmill and therefore not sport-specific
Yoshida et al. 2002 [85]	Baseball	*n* = 7, 21 y male college players	Sports drink (3.6% CHO) consumed at a volume equivalent to 20, 40, 60, and 80% of sweat losses during a usual practice (3.8 h) 10 s maximal anaerobic power output during cycling measured before and after baseball practice Env conds: 29.2 °C WBGT	−3.9% (20% replaced), −2.5% (40% replaced), −1.7% (60% replaced), −0.7% (80% replaced)	NA	Maximal anaerobic power decreased (by ~13% from baseline) with −3.9% ∆BM NS: change in maximal anaerobic power with −0.7%, −1.7%, or −2.5% ∆BM	Subjects not blinded to hydration status Practice sessions not standardized between trials Potential confounding effect of CHO

Values are means or ranges where specified

*BM* body mass, *CHO* carbohydrate, *Env conds* environmental conditions, *EUH* euhydration, *GPS* global positioning system, *HR* heart rate, *HYPO* hypohydration, *LIST* Loughborough Intermittent Shuttle Test, *mph* miles per hour, *NA* not available, *NS* no significant effect, *RH* relative humidity, *RPE* rating of perceived exertion, *Tc* body core temperature, *WBGT* wet bulb globe temperature

Although racket sports (e.g., tennis) are typically considered individual sports, they are included in this review because they are team sports when played in “doubles” competition (a match between two pairs of players) and because of the intermittent high-intensity physical demands and technical skill requirements. Sports that require skill but do not rely heavily upon intermittent bouts of high-intensity running (e.g., golf) are not discussed here. Endurance, strength/power, combat, and esthetic sports are also outside the scope of this review.

### Fluid Balance Terminology

Body fluid balance is primarily a function of an individual’s fluid intake (i.e., hydration practices) relative to his or her fluid losses (i.e., sweat) during exercise. The term “euhydration” refers to maintenance of “normal” baseline body water content, while the terms “hypohydration” and “hyperhydration” refer to body water deficits and excesses beyond euhydration, respectively. The term “dehydration” is defined as the process of the dynamic loss of body water or the transition from euhydration to hypohydration. The simplest method to assess an individual’s acute change in hydration status (or fluid balance) is to compare his/her body mass to baseline values [13]. For example, 3% hypohydration is defined as a water deficit equal to 3% BML. It is acknowledged that a small portion of body mass loss during exercise occurs due to substrate oxidation, that is, non-water mass loss through expiration of carbon dioxide. The reader is referred to other papers that discuss potential errors in hydration assessment methodologies in greater detail [13, 14]. Importantly, the BML method of hydration assessment and related terminology have been used in all relevant studies identified for the aims of this review and, therefore, will be used throughout the discussion that follows.

## Fluid Balance in Team Sports

**Fig. 1:**
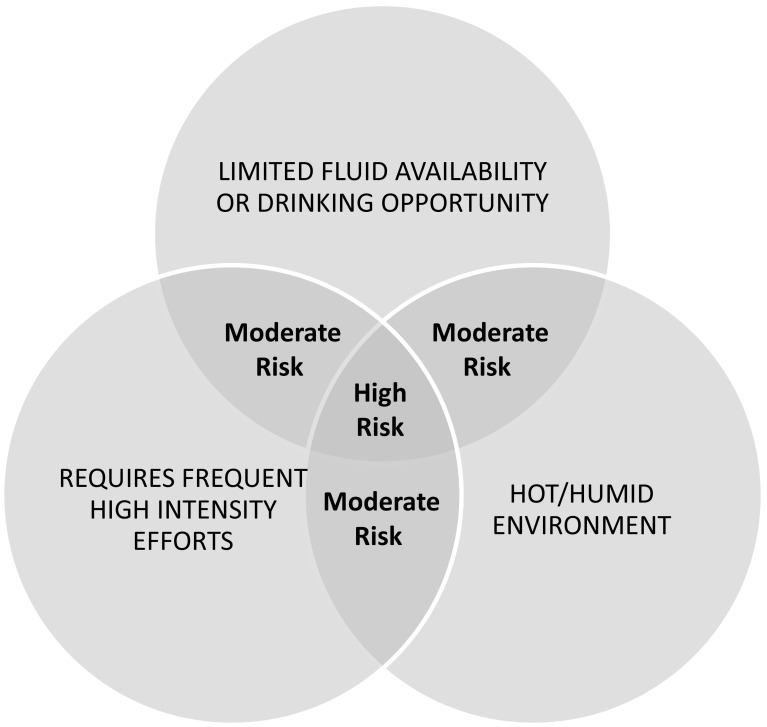
Venn diagram showing risk levels for the development of significant hypohydration (>2% body mass loss). The factors shown in this diagram elevate risk of hypohydration by increasing sweat loss (intensity and environment) or limiting fluid replacement. Note that “hot/humid environment” can include wearing protective equipment (which would create a hot/humid microclimate via encapsulation), as well as hot/humid ambient conditions. This diagram applies to the typical duration of team sports practice/competition, which is generally 1–2 h. In instances of shorter or longer duration, risk level may decrease or increase, respectively. Various team sports can be generally classified into low, moderate, and high risk based upon how the structure/rules of the game impact the three risk factors. However, on an individual basis, risk of hypohydration may shift to a lower or higher category depending upon various factors such as drinking behavior (e.g., cultural/social factors), playing position (e.g., soccer goalie vs. midfielder, or baseball catcher vs. outfielder), and playing time (e.g., reserve vs. starter). In addition, for outdoor sports the risk may shift depending upon time of day and season of the year that training/competition takes place (i.e., due to differences in temperature/humidity throughout the day/year)

Figure 1 shows a Venn diagram illustrating risk levels for the development of significant hypohydration (>2% BML). Intuitively, the factors that elevate risk of hypohydration are those that increase thermoregulatory sweat loss (hot/humid environment and high exercise intensity) or limit fluid replacement (low availability of fluid or opportunity for drink breaks). Sweating rate and/or fluid balance has been researched the most in soccer [15–35], followed by American Football [36–48], tennis [49–58], basketball [15, 59–66], rugby [67–73], and ice hockey [74–78]. Australian Rules Football [79, 80], cricket [81–83], baseball [84, 85], futsal [17], Gaelic Football [86], netball [15], beach volleyball [87], court volleyball [88], field hockey [89], badminton [90], and water polo [91] have been studied to a lesser extent. Table 1 provides a summary of the findings by sport organized per level of risk for hypohydration according to factors identified in Fig. 1.

In soccer, a broad range in mean sweating rate has been reported (0.3–2.5 L/h). This is due, in part, to the varied environmental conditions (5–43 °C) and athlete characteristics, and likely accounts for the considerable variability in fluid balance (0.4% body mass gain to 3.5% BML) across studies. Significant hypohydration (>~2% BML) has been reported in soccer, particularly in high-caliber players during match play in the heat [16, 18, 20–22]. The combination of high sweating rates and infrequent opportunities to drink can make it difficult to maintain fluid balance in soccer. Of note, the International Federation of Association Football recently made a rule change that allows for water breaks in extreme environmental conditions (wet bulb globe temperature, WBGT >32 °C) to combat this challenge [92].

High mean sweating rates have been reported in American Football (0.6–2.9 L/h), with the large body size of players partially responsible [4, 8, 40, 41], as well as the equipment/uniform requirements [6, 7]. In addition, all studies in American Football have been conducted during preseason training, which typically occurs in warm–hot summer weather [3]. Despite high sweating rates, the observed disturbances in fluid balance have generally been mild [3], with the exception of one study [36] reporting a mean BML of ~2.3–2.4% (SD: 1.1–1.9%) in players practicing in full pads under high levels of heat stress (29–32 °C WBGT). Similar results have been reported in rugby and Australian Football, where high sweating rates led to significant hypohydration in some [67, 80] but not all studies [67, 70]. High mean sweating rates have also been reported in basketball [15, 59, 64, 65], tennis [50, 51, 58], ice hockey [74–76], and beach volleyball [87]; however, mean BML was <2%, suggesting that drinking opportunities were sufficient to provide most athletes with enough fluid to offset sweat losses.

Some studies have tested athletes multiple times to determine intra-individual variability in sweating rates and fluid balance. For example, Australian Rules Footballers exhibited higher sweating rates and accrued greater levels of hypohydration during high-intensity training and game simulations (1.1–1.3 L/h and 3.4–3.5% BML) compared with low-intensity training (0.8 L/h and 2.1% BML) [34]. Similarly, studies in soccer [16, 22] and tennis [58] have reported that warm–hot conditions elicited higher sweating rates during match play than cool–temperate environments. While higher rates of sweat loss can lead to greater body mass deficits [22, 58], this is not always the case [15, 16]. For example, Mohr et al. [16] found that soccer players accrued the same level of hypohydration (1.8 and 1.9% BML, respectively) during indoor (21 °C) and outdoor (43 °C) match play despite significantly different sweating rates (1.6 vs. 2.5 L/h, respectively). Taken together, it seems that the sports or conditions associated with higher sweating rates are not always associated with the higher levels of hypohydration. Factors related to fluid availability (type and amount), drinking opportunities (per the rules and structure of the game), exercise duration, hydration education, and personal preferences also play an important role in determining fluid balance [11].

## Literature Review of Hypohydration and Performance

### Cognition

#### Soccer

Three studies have investigated hypohydration and cognitive performance in soccer [93–95]. Overall, these studies suggest that fluid restriction has minimal effects on cognition, at least up to 2.5% BML. For instance, Edwards et al. [94] reported no differences in mental concentration (number identification test) when male soccer players drank water (0.7% BML) versus water mouth rinse (2.1% BML) or fluid restriction (2.4% BML) trials. Similar results were found in a separate study comparing the effects of fluid intake (1.4% BML) versus no fluid intake (2.4% BML) on mental concentration (number identification test) in male semiprofessional soccer players [95].

Bandelow et al. [93] used a field study approach and complex data modeling to determine the relative contribution of various factors, including BML, on cognitive performance in male university soccer players. In this study two trials were completed; players drank water ad libitum in the first match and were encouraged to drink a sports drink and/or water in the second match. Matches were played in a hot environment (34 °C, 64–65% relative humidity). Before, at halftime, and after each match the players completed a battery of cognitive tests, which included fine motor speed (finger tapping test), visuomotor reaction time (visual sensitivity test), visuospatial working memory (Corsi block test), and working memory simple reaction time (Sternberg test). Bandelow et al. [93] reported that hypohydration (up to 2.5% BML) impaired working memory reaction time, but had no effect on any other measure of cognitive performance. Instead, maintenance of blood glucose (presumably from sports drink consumption) and core temperature changes played more important roles in determining speed and accuracy during the cognitive battery [93].

#### Basketball

Two basketball studies have tested the impact of hypohydration on cognitive performance, with mixed results [96, 97]. Hoffman et al. [96] found no differences in visual reaction time during a hand-eye reaction test (Dynavision D2) when female college players drank water versus no fluid to accrue 2.3% BML. By contrast, the number of successful attempts during a lower body reactive agility test (Quick Board™) was significantly lower in the no-fluid trial [96]. In addition, Baker et al. [97] found that hypohydration was associated with impaired vigilance in male players. In this study, subjects performed the test of variables of attention at baseline, after exercise-heat stress to induce 1–4% hypohydration or maintain euhydration (i.e., pre-game), and then after a simulated basketball game (where target levels of BML were maintained). The players made significantly more errors of omission and commission and had slower response times (by ~6–8%) in the 1–4% BML trials than the euhydration trial [97]. In this study there were no differences in vigilance between the graded levels of hypohydration [97].

#### Field Hockey

The effect of hypohydration on cognitive performance has been tested in one field hockey study by MacLeod and Sunderland [98]. On day 1 of this study, elite female field hockey players underwent 2 h of passive heat stress to stimulate fluid loss. After completion of this dehydration phase, subjects’ fluid intake was controlled such that the following morning (day 2) they were either euhydrated or 2% dehydrated. On day 2, players completed a field hockey skill test [99] before and after a 1-h intermittent treadmill protocol in which players drank water ad libitum (maintained BML difference between trials). Decision-making time during the skill test was 7% slower in the 2% hypohydrated versus the euhydration trial, but only before (not after) the intermittent exercise [98]. Thus the effects of hypohydration on cognitive performance seem to be inconsistent in this study [98]. Nonetheless, it is interesting to note that the study by MacLeod and Sunderland [98] has been the only one to employ a cognitive test that is sport-specific as opposed to others (discussed above in the Sects. 4.1.1 and 4.1.2) that used tests originally designed for the general population.

#### Multiple Sports

In a study of college lacrosse and American Football players, D’Anci et al. [100] compared the effects of no fluid versus water intake (euhydration) during 60- to 75-min high-intensity practices on performance during a subsequent cognitive test battery. The players accrued 1.8% BML and 1.2% BML in the no-fluid trials of study 1 and study 2, respectively. Vigilance performance was impaired by 3–4% when athletes were 1.8% hypohydrated, but was not impacted by 1.2% hypohydration. None of the other measures of cognition in the test battery (e.g., memory, reaction time, visual perception, math, and map planning) were affected by hydration status in either study.

#### Summary

Based on the results of seven studies completed to date, the impact of hypohydration (most studies involved ~1 to ~2.5% BML) on cognitive performance of team sport athletes is equivocal. In four studies, vigilance, decision-making time, working-memory reaction time, or reactive agility were impaired with hypohydration; however, across all studies no other measure of cognition (e.g., mental concentration, fine motor speed, visual perception, visuomotor reaction time, math) was affected. This inconsistency is likely due in part to the aspects of cognition measured, types of cognitive tests used, the reliability and sensitivity of these tests, and other factors related to study design. Cognitive performance is difficult to measure, particularly in the context of sports, and very few studies have employed tests directly relevant to team sports performance. More work is needed to develop and validate sport-specific cognitive performance assessments.

### Sport-Specific Skills

#### Basketball

The potential impact of hypohydration on basketball shooting performance has been assessed in six studies [60, 63, 96, 101–103]. Most of these studies have investigated ~2% hypohydration and found mixed results. For instance, Hoffman et al. [96, 101] found no impact of fluid restriction (1.9 and 2.3% BML) versus water intake on shooting performance by male youth [101] or female college players [96]. However, it is interesting to note that field goal percentage decreased by 8.1% (not statistically significant) from the first to second half in the no-fluid trial, but was maintained (1.6% increase) when male youth players were allowed to drink water [101]. Similar results were found in a study comparing the effects of no fluid (2.5% BML) versus ad libitum water intake (1.1% BML) in male youth players; Carvalho et al. [60] found no significant differences in shooting performance between conditions, but did report a non-significant 5.8% lower two-point field goal accuracy with no fluid. In another study with male youth players, Dougherty et al. [103] reported that 2% hypohydration was associated with significantly lower shooting percentage (by 8% combined for all shots) compared with euhydration.

In a descriptive study, Brandenburg and Gaetz [63] allowed elite female players unlimited access to the drink of their choice (sports drink and or water) during two international games. In both games the players accrued up to ~2.0% BML. The authors reported a significant inverse relation between BML and field goal percentage (*r* = −0.61) in the second game, but no relation in the first game. While measuring the impact of hydration status on performance during actual game play increases the ecological validity of this study, the interpretation of results is limited due to the potential impact of confounding factors (e.g., concomitant carbohydrate ingestion, additional dietary intake behaviors, and defensive prowess by the opposing team).

The effects of graded levels of hypohydration on basketball shooting performance have been tested in one study of male players. Baker et al. [102] found that, compared with euhydration, increasing levels of hypohydration (2–4%) led to a progressive 7–12% decrease in the total number of shots made during a simulated game. However, there were no differences in shooting percentage between trials. In this study, players were allotted a standardized time to make as many shots as possible during each drill. Thus, the decrease in shots made with hypohydration were partially a result of fewer shot attempts due to slower sprinting and dribbling speeds between shots [102]. Taken together, the results of the six studies in basketball suggest that ≥2% hypohydration can potentially impact shooting performance, perhaps due to decreasing shooting accuracy and/or slowing the frequency of shot attempts. Both factors can impact the total number of points scored, which plays an important role in dictating the outcome of a basketball game [104].

#### Soccer

Three studies [95, 105, 106] have tested the impact of fluid restriction on soccer-specific skills performed before, during, and after a 90-min intermittent protocol (Loughborough intermittent shuttle running test; LIST [107]). Ali et al. [106] reported no impact of fluid restriction (2.2% BML) versus water intake (1.0% BML) on passing performance [108] in female Premier division players. Similarly, Owen et al. [105] found that passing and shooting skills [108] of male semiprofessional players were unaffected by fluid restriction (2.5% BML) compared with ad libitum (1.1% BML) or prescribed (0.3% BML) water intake. By contrast, and also in semiprofessional soccer players, McGregor et al. [95] found a 5% deterioration in dribbling skill during the LIST with no fluid ingestion (2.4% BML) while skill performance was maintained during the fluid intake trial (1.4% BML). Taken together, these results suggest that the effect of hypohydration on soccer performance may be dependent upon the type of skill measured, albeit more research is needed and, in particular, with cohorts varying in competitive level.

#### Cricket

The effect of hypohydration on skill in well-trained cricket players has been investigated in two studies [83, 109]. Devlin et al. [109] compared the effects of fluid restriction (2.8% BML) versus prescribed fluid intake (0.9% BML) during 1-h intermittent exercise-heat stress (28 °C) on performance of a subsequent bowling skill test. Compared with prescribed fluid intake, fluid restriction was associated with a ~15–16% impairment in bowling accuracy, but had no effect on bowling velocity. Recently, Gamage et al. [83] found that fluid restriction (3.7% BML) during 2-h cricket training in the heat (27–33 °C, 66–89% relative humidity) led to a 1–7% decrease in speed and 14–22% decrease in accuracy of bowling [110] and throwing [111] tests, whereas performance during the cricket skill test was maintained in the fluid provision trials (0.9% BML) [83].

#### Field Hockey

One study has investigated the potential effects of previous day passive heat stress-induced hypohydration on field hockey skill. MacLeod and Sunderland [98] employed a test involving dribbling, passing, and shooting at an illuminated target after a 60-min intermittent treadmill protocol with ad libitum drinking [99] and found no impact of 2% hypohydration versus euhydration on field hockey-specific skill in elite female players.

#### Tennis

There are limited data available on the effects of hypohydration on skill performance in racquet sports. One study has reported no differences in post-match tennis shot accuracy during a ball machine test when male and female players drank water (1.1% BML) or no fluid (2.7% BML) during a 2-h simulated tennis match [112].

#### Summary

The effect of hypohydration on skill performance seems to be inconsistent across sports. Studies suggest that ~2–4% hypohydration can impair shooting performance in basketball and bowling/throwing in cricket. By contrast, the balance of studies suggests a minimal impact of ~2–3% hypohydration on skill performance in soccer, field hockey, and tennis. Like cognition, skill is difficult to measure, and more work is needed to develop reliable, valid, and sensitive sport-specific tests to use in future studies investigating the impact of hypohydration on skill.

### Sprinting

#### Basketball

The impact of hypohydration on sprint performance has been assessed in three basketball studies [60, 102, 103]. During a simulated basketball game with male players, increasing levels of hypohydration (2, 3, and 4% BML) were associated with progressively longer (i.e., sprint performance was decreased) total sprint times (by 7, 8, and 16%, respectively) [102]. In a study of youth male players, hypohydration (2% BML) led to a significant 6% longer total and mean sprint time throughout a simulated game compared with euhydration [103]. However, there was no effect of fluid restriction (2.5% BML) versus ad libitum water intake (1.1% BML) on sprint performance after training in another study of youth male basketball players [60].

#### Soccer

Three studies have tested the impact of hypohydration on 15-m sprint performance in soccer players during the LIST protocol [95, 106, 113]. Ali et al. [106] reported no difference in mean sprint time between trials in which female Premier division soccer players drank water (1.0% BML) or no fluid (2.2% BML) throughout the LIST protocol. In a similar study design with male university soccer players, Ali and Williams [113] reported no impact of water restriction (3.7% BML) versus water ingestion (2.3% BML) on mean 15-m sprint time. On the other hand, McGregor et al. [95] found that the mean 15-m sprint time of male semiprofessional soccer players was significantly longer in the last 15-min block of the LIST protocol when fluid was withheld (2.4% BML) versus when allowed to drink (1.4% BML) during the 90-min LIST.

#### Batting Sports

Hypohydration and sprint performance in baseball and cricket have been investigated in two studies [83, 114]. In college baseball players, hypohydration by 3% BML (induced by previous day exercise-heat stress) was associated with a significant 3–4% longer mean time to complete 30-m sprints during the latter bouts of an intermittent sprinting protocol [115] compared with euhydration [114]. In a study of male elite cricketers, Gamage et al. [83] also found impaired sprinting performance as a result of hypohydration. Sprint time increased significantly (by 2.2%) when fluid was restricted to 4 ml/kg/h (3.7% BML) throughout 2 h of cricket training, whereas performance was maintained from pre- to post-training when 12–15 ml/kg/h fluid was provided (0.9% BML) [83].

#### Summary

The effect of hypohydration on sprint performance has been measured in eight studies across four sports. For soccer, the balance of the literature suggests that ~2–4% hypohydration is unlikely to impact mean 15-m sprint performance throughout an entire bout of 90-min intermittent exercise, but may prolong sprint time in the latter stages (e.g., last 15 min of a 90-min session). Results are more consistent in basketball and batting sports, with most studies reporting longer time to complete sprints when athletes are hypohydrated by ~2–4% in basketball or ~3–4% in baseball and cricket. More research is needed to determine the impact of hypohydration on sprint performance in other team sports.

### Sport-Specific Lateral Movements

#### Basketball

Three studies have employed similar lateral slide drills to simulate defensive movements in basketball [60, 102, 103]. Baker et al. [102] reported that the time to complete defensive slide drills by male players throughout a simulated game was not impacted by 1–2% hypohydration, but was significantly longer with 3–4% hypohydration compared with euhydration. In another study, Dougherty et al. [103] found that 2% hypohydration was associated with ~7% longer defensive slide times in youth male players throughout a simulated game. However, Carvalho et al. [60] found that defensive slide times after training were not different between fluid restriction (2.5% BML) and ad libitum water intake (1.1% BML) trials in male youth players.

#### Summary

The ability to make quick lateral movements is important for performance in many sports, such as defensive sliding in basketball, fielding in baseball, or returning a groundstroke in tennis. However, the effect of hypohydration on performance of sport-specific lateral movements has only been tested in basketball. The mixed results reported in these studies suggest that the impact of hypohydration, ranging from ~1 to 4% BML across studies, is currently unclear. More research on sport-specific lateral movement performance is needed in basketball and other relevant team sports.

### Vertical Jump Height and Anaerobic Power

#### Basketball

Four basketball studies have investigated the effects of hypohydration on jumping performance, including maximal jump height [96, 101–103], time to complete a set number of jumps [102, 103], and peak or mean anaerobic power during repeated jump tests [96, 101]. These studies report no impact of hypohydration (~1–4% BML) on maximal jump height [101–103]. However, Baker et al. [102] reported significantly longer repeated jump time with 4% hypohydration versus euhydration. In addition, Hoffman et al. [101] found that post-game anaerobic power [116] was 19% lower when fluid was restricted (1.9% BML) versus when water intake was permitted, although this difference did not reach statistical significance.

#### Baseball

One study has measured the effect of graded dehydration on anaerobic power in college baseball players. In a cross-over study, Yoshida et al. [85] induced 0.7, 1.7, 2.5, and 3.9% BML in players during a 3.8-h practice in the heat (29° WBGT) by having them drink to replace 80, 60, 40, and 20% of fluid losses, respectively. Maximal anaerobic power during a 10-s cycling test was decreased significantly by ~13% from pre- to post-exercise with 3.9% BML, but there was no significant change with the lower levels of hypohydration.

#### Tennis

One study has compared the effects of ingesting water (1.1% BML) or no fluid (2.7% BML) during a 2-h simulated match on maximal jump height and anaerobic power (Sargent jump test) in male and female tennis players. In this study, Burke and Ekblom [112] found no change in performance from pre- to post-practice with either fluid intake condition.

#### Summary

Jump height and anaerobic power are critical to performance in many team sports; however, only six studies have measured the potential effects of hypohydration in sport-specific studies. These studies suggest that hypohydration is unlikely to have a negative impact on vertical jump height. However, anaerobic power may be impaired by hypohydration, especially at higher levels of hypohydration (~4% BML). In general, these results are in agreement with recent reviews and meta-analyses on the effect of hypohydration on jumping ability and anaerobic power [12, 117]. Nonetheless, more research is needed to understand how hypohydration may impact jump height and anaerobic power in the context of team sport performance. Finally, while a recent meta-analysis concluded that ~3% BML may improve body mass-dependent tasks such as vertical jumping ability [117], this has not been found in the team sport studies reviewed here. As demonstrated by Cheuvront et al. [118], the theoretical improvement in jump height associated with a dehydration-induced body mass deficit may be offset by an inability to produce the same degree of contractile force, thus confounding the interpretation of how hypohydration affects body mass-dependent tasks.

### Intermittent High Intensity Running Capacity

#### Soccer

Two studies [94, 105] have employed the Yo–Yo intermittent recovery test [119, 120] to determine the effect of hypohydration on intermittent running capacity in soccer. Owen et al. [105] measured performance during the Yo–Yo test in male semiprofessional soccer players before and after they completed the LIST protocol. There were no differences in Yo–Yo performance between trials in which players drank no fluid (2.5% BML), water ad libitum (1.1% BML), or water to replace ~90% of fluid losses (0.3% BML) during the LIST protocol. By contrast, in another study, 13–15% less distance was covered during the Yo–Yo test when male soccer players were 2.1% (water mouth rinse) and 2.4% (no fluid) hypohydrated versus when they were allowed water ingestion (0.7% BML) during 45 min of cycling followed by a 45-min match [94].

#### Cricket

In a study of male, well-trained bowlers, Devlin et al. [109] compared the effect of fluid restriction (2.8% BML) versus prescribed fluid intake (0.5% BML) during 1 h of intermittent exercise-heat stress on subsequent performance of a maximal multi-stage shuttle run [121]. Intermittent running capacity was significantly impaired when fluid was restricted, as the bowlers completed 7.7% fewer shuttles in the 2.8% BML versus 0.5% BML trial.

#### Summary

For many team sports, the capacity to sustain high intensity efforts alternated with rest or lower intensity periods throughout a game is critical to the success of an athlete. To date, two out of three studies have found a detrimental effect of 2–3% hypohydration on intermittent running capacity. However, more research is needed, particularly on the sports that are highly dependent upon intermittent running capacity (e.g., soccer, rugby, field hockey, and basketball).

## Potential Mechanisms and Modifying Factors for the Effects of Hypohydration on Performance

### Overview of Physiological Effects of Hypohydration During Exercise

Because sweat is hypotonic compared with plasma [122], exercise-induced hypohydration is associated with an increase in plasma osmolality and a decrease in plasma volume (i.e., hyperosmotic hypovolemia). Hypovolemia results in a decrease in stroke volume and a compensatory increase in heart rate to maintain a given cardiac output [123–125]. Hypovolemia and hyperosmolality delay the onset and decrease the sensitivity of the sweating and skin blood flow responses to hyperthermia [126–128], thus increasing heat storage [125, 129]. Consequently, exercise performance that is dependent upon the cardiovascular and thermoregulatory systems, such as aerobic exercise in the heat, can be impaired by hypohydration [9, 12]. The physiological mechanisms underlying the effect of hypohydration on aerobic performance have been well studied (for reviews, see Sawka et al. [130, 131] and Cheuvront et al. [132]). By contrast, much less is known about the potential mechanisms for the detrimental effects of hypohydration on team sport performance. The next section summarizes the proposed mechanisms by which hypohydration could impair cognition, technical skill, and physical performance related to team sports.

### Cognition

The effect of hypohydration on cognition has been widely researched. While decrements in cognitive performance with hypohydration have been reported in some studies of athletes [97, 98, 100, 133], healthy young adults [134–138], and military personnel [139, 140], other studies have found no effect of hypohydration [94, 95, 141–146]. Furthermore, a clear mechanism by which hyperosmolality or hypovolemia per se would impair cognition is currently lacking (for a review, see Cheuvront & Kenefick [12]). In brief, hypohydration has been suggested to mediate decrements in brain function by decreasing cerebral blood flow, reducing brain volume, or increasing blood–brain barrier permeability. However, a consistent effect of hypohydration on these measures of brain function [147–153] at the levels of BML typically reported in the cognition literature (e.g., 1–4% BML in team sport studies) has not been found.

An alternative explanation, previously described by Cheuvront and Kenefick [12], is that symptoms of hypohydration, such as thirst, headache, or negative mood states (e.g., fatigue), may distract subjects during cognitive tasks and subsequently impair performance. Moreover, individual variability in cognitive resiliency (ability to overcome the stressors of hypohydration) may explain, in part, the equivocal findings in the cognition literature [12]. For example, Szinnai et al. [145] found that 2.6% BML induced by water deprivation had no impact on cognitive-motor function, but significantly increased ratings of perceived effort and concentration necessary for test completion. Furthermore, Kempton et al. [147] showed that although mild hypohydration did not impair cognitive performance or cerebral perfusion, higher levels of neuronal activity (as indicated by a greater increase in the fronto-parietal blood oxygen-level-dependent response) were required to perform an executive function task. Thus, it may be that some individuals are better at increasing concentration sufficient to overcome symptomologic distracters of hypohydration and achieve the same level of performance as that of a euhydrated state [12]. In the team sport literature reviewed in Sect. 4.1, hypohydration consistently increased ratings of thirst, perceived exertion, and fatigue, but subsequent effects on cognitive performance were equivocal.

### Physical Performance

In team sports high-intensity efforts are performed within the context of intermittent exercise over a prolonged period of time (1–2 h). Thus, it is plausible that reductions in aerobic capacity [154–157] or muscle endurance [117, 158] that have been shown to occur with hypohydration, could help explain the impaired physical performance (sprinting, lateral movements, and intermittent running capacity) reported in studies mimicking the demands of team sports training/play. In addition, hypohydration has been shown to result in decreased muscle blood flow [159, 160] and alterations in skeletal muscle metabolism (increased lactate, muscle glycogenolysis, and carbohydrate oxidation) [160–163]. However, to date these findings have mostly been documented in prolonged cycling exercise, which is likely a result of the difficulty in obtaining invasive physiological measurements in team sport athletes on the field of play. To our knowledge, only one team sport performance study has measured the effect of hydration status on markers of muscle metabolism. Ali and colleagues [106] found that blood lactate concentration was significantly higher (7.2 vs. 3.7 mmol/L) when female soccer players drank no fluid (2.2% BML) compared with when they ingested fluid during the 90-min LIST protocol (1.0% BML). However, in this study, sprint and skill performance were not impacted by hydration status [106]. It is also interesting to note that, in the studies reviewed (Tables 2, 3), performance was no more likely to be impaired in sports with high aerobic demands (e.g., soccer) than sports that have more rest opportunities (e.g., basketball) or are lower intensity (e.g., baseball, cricket). This is somewhat surprising given the reported detrimental effects of hypohydration on endurance performance [9, 12], but it is likely that study limitations and various modifying factors play a role in the discrepancy in these findings (discussed in more detail in Sect. 5.5).

Another potential mechanism to consider is the hyperthermic effect of hypohydration, as some sport-specific studies have reported higher body core temperatures with fluid restriction versus fluid intake [94, 102, 103]. Drust et al. [164] reported that elevated core and muscle temperatures during a 40-min intermittent cycling protocol were associated with impaired repeated sprint performance. The authors concluded that the results may be related to the influence of hyperthermia on central nervous system function. Central fatigue, as indicated by an impaired ability to sustain maximal muscle activation during sustained contractions, has been implicated in exercise performance decrements associated with hyperthermia [165–167]. It is thought that multiple factors (including core and skin temperature) likely provide afferent inputs for central nervous system integration and reduce motor drive to skeletal muscles. The reader is referred to a review by Nybo et al. [165] for a recent comprehensive discussion on physiological factors governing hyperthermia-induced fatigue. It is important to note that while hyperthermia (increased core temperature) can impair performance during prolonged exercise, increased muscle temperature could enhance certain aspects of physical performance [168]. In particular, improved sprinting performance has been reported in soccer [16, 169], perhaps as a result of improved muscle contractile properties and anaerobic power [170, 171], in hot versus temperate environments. However, these changes occur irrespective of hypohydration, as Mohr and colleagues [16] found faster peak sprinting speed in hot versus temperate conditions when players accrued similar levels of BML between trials with ad libitum fluid intake (1.9 and 1.8%, respectively). As such, optimal performance strategies may involve both maintenance of muscle temperature as well as the limitation of excessive hypohydration.

Finally, as with cognition, it is possible that psychological factors are also involved in hypohydration-induced decrements in physical performance. Negative mood states and other stressors associated with fluid restriction may distract athletes from giving their full effort toward performing the high-intensity exercise task. In support of this notion, most (10 of 11) team sport studies that measured subjects’ perceived exertion or fatigue found that ratings were significantly elevated in conditions of fluid restriction versus fluid intake (see Table 3). In addition, there may be some interplay between familiarization with hypohydration, perceived exertion, and the effect of hypohydration on performance. Although not specific to team sports, Flemming and James [172] reported some support for this concept in recreationally active men. In this study, 2.4% BML impaired 5-km treadmill running performance (by 5.8%) when subjects were unfamiliar with the hypohydration protocol. However, there was an attenuation of subjects’ ratings of perceived exertion and the performance decrement (1.2%) after completion of four familiarization sessions designed to habituate subjects with the hypohydration protocol. While these novel data are interesting, more research is needed before definitive conclusions can be made regarding the effect of familiarization with hypohydration on ratings of perceived exertion and performance.

### Sport-Specific Technical Skills

The execution of sport-specific skill is a complex process, as it is dependent upon several aspects of physical and cognitive function. For example, a successful shot attempt during a basketball game requires a combination of fine motor (ball control) and gross motor (balance and coordination) skills, physical abilities (power, strength, and speed), concentration, and decision-making skills, among other factors. Thus, if hypohydration impairs cognition or physical performance, either directly through hyperosmotic/hypovolemia-induced changes in physiological function or as a byproduct of the distracting symptoms of hypohydration, then these mechanisms could also account for impaired execution of technical skills.

Other proposed mechanisms include changes in vestibular function, as some studies have reported impaired postural balance (increased body sway) with hypohydration after exercise [173–176]. However, other studies have reported no impact of hypohydration on balance control [133, 141, 177]. Theoretically, balance is more likely to be impaired when hypohydration is combined with hyperthermia [175] or fatigue from previous exercise [173, 174]. However, Seay et al. [177] reported no relation between standing balance and levels of hypovolemia or hyperosmolality. As such, a clear physiological mechanism by which hypohydration could impair postural control has not yet been identified.

Future studies are needed to elucidate which physiological mechanisms or combination thereof may account for the detrimental impact of hypohydration on cognition, technical skill, and physical performance reported in some studies. For more details on potential mechanisms underlying the impact of hypohydration on various aspects of performance the reader is referred to previous reviews [12, 130, 132].

### Modifying Factors

Many methodological differences among studies likely contribute to the inconsistent results reported across the literature. For example, the subject characteristics (e.g., sex, age, caliber of athlete), method of dehydration (e.g., passive heat, exercise, or exercise-heat stress), and/or BML differences between hypohydration and control trials varied considerably among studies. Thus, a relevant question that follows is: Do certain factors modify the impact of hypohydration on performance (i.e., are there interaction effects)? To date, no studies have addressed this question directly. However, when comparing studies across the literature (see Table 4) there seems to be no clear pattern regarding the impact of sex, age, or athlete caliber on the effects of hypohydration on team sport performance. This is due in part to the limited data available, as only six studies have included female subjects [63, 96, 98, 100, 106, 112] and only three studies have tested youth athletes [60, 101, 103]. A broad range of athlete calibers have been tested across studies with equivocal results within caliber (see Table 4), suggesting that a particular level of athlete is not more or less likely to be negatively affected by hypohydration based on the currently available data in team sports. However, studies directly comparing team sport athletes with different skill levels are needed.

**Table 4:** Modifying factors for the effect of hypohydration on performance in team sports studies

	Sex		Age		Athlete caliber		
	Male	Female	Youth	Adult	Rec and/or comp	College	Semipro, pro, and/or elite
Cognition	3/5 (60%)	3/3 (100%)	–	5/7 (71%)	1/2 (50%)	3/3 (100%)	1/2 (50%)
References	[93–95, 97, 100]	[96, 98, 100]	–	[93–98, 100]	[94, 97]	[93, 96, 100]	[95, 98]
Skill	5/9 (56%)	1/5 (20%)	1/3 (33%)	5/10 (50%)	2/4 (50%)	1/2 (50%)	3/7 (43%)
References	[60, 83, 95, 101–103, 105, 109, 112]	[63, 96, 98, 106, 112]	[60, 101, 103]	[63, 83, 95, 96, 98, 102, 105, 106, 109, 112]	[101–103, 112]	[96, 109]	[60, 63, 83, 95, 98, 105, 106]
Sprint	5/7 (71%)	0/1 (0%)	1/2 (50%)	4/6 (67%)	2/2 (100%)	1/2 (50%)	2/4 (50%)
References	[60, 83, 95, 102, 103, 113, 114]	[106]	[60, 103]	[83, 95, 102, 106, 113, 114]	[102, 103]	[113, 114]	[60, 83, 95, 106]
Lateral movements	2/3 (67%)	–	1/2 (50%)	1/1 (100%)	2/2 (100%)	–	0/1 (0%)
References	[60, 102, 103]	–	[60, 103]	[102]	[102, 103]	–	[60]
Jumping/power	2/5 (40%)	0/2 (0%)	0/2 (0%)	2/4 (50%)	1/4 (25%)	1/2 (50%)	–
References	[85, 101–103, 112]	[96, 112]	[101, 103]	[85, 96, 102, 112]	[101–103, 112]	[85, 96]	–
Intermittent high intensity running capacity	2/3 (67%)	–	–	2/3 (67%)	1/1 (100%)	1/1 (100%)	0/1 (0%)
References	[94, 105, 109]	–	–	[94, 105, 109]	[94]	[109]	[105]

	Method of dehydration			BML difference between control (fluid intake) and hypo (fluid restriction) trials			
	Passive heat stress	Exercise only	Exercise + heat stress	1–2%	3–4%		
Cognition	1/1 (100%)	2/4 (50%)	2/2 (100%)	4/6 (67%)	1/1 (100%)		
References	[98]	[94–96, 100]	[93, 97]	[94–98, 100]	[97]		
Skill	0/1 (0%)	2/8 (25%)	4/4 (100%)	4/12 (33%)	2/2 (100%)		
References	[98]	[60, 63, 95, 96, 101, 105, 106, 112]	[83, 102, 103, 109]	[60, 63, 95, 96, 98, 101–103, 105, 106, 109, 112]	[83, 102]		
Sprint	–	1/4 (25%)	4/4 (100%)	3/6 (50%)	3/3 (100%)		
References	–	[60, 95, 106, 113]	[83, 102, 103, 114]	[60, 95, 102, 103, 106, 113]	[83, 102, 114]		
Lateral movements	–	0/1 (0%)	2/2 (100%)	1/3 (33%)	1/1 (100%)		
References	–	[60]	[102, 103]	[60, 102, 103]	[102]		
Jumping/power	–	0/3 (0%)	2/3 (67%)	0/6 (0%)	2/2 (100%)		
References	–	[96, 101, 112]	[85, 102, 103]	[85, 96, 101–103, 112]	[85, 102]		
Intermittent high intensity running capacity	–	1/2 (50%)	1/1 (100%)	2/3 (67%)	–		
References	–	[94, 105]	[109]	[94, 105, 109]	–		

Values are number of studies reporting a detrimental effect of hypohydration on performance out of total number of studies with the percentage shown in parenthesis

Dashes indicate no studies available

*BML* body mass loss, *Comp* competitive, *Hypo* hypohydration, *Pro* professional, *Rec* recreational, *Semipro* semiprofessional

As shown in Table 4, one of the factors that does seem to modify the impact of hypohydration on performance in team sport athletes is the method of dehydration. When dehydration was induced via exercise in the heat, subsequent performance was usually impaired with respect to cognition, skill, sprinting, lateral movements, jumping/power, and intermittent running capacity (the only exception was jumping performance in one study [103]). By contrast, when dehydration was induced via exercise alone, subsequent performance was impaired in ≤ 50% of the studies within each of the performance categories. This finding is perhaps not surprising given the well-established deleterious effect of environmental heat stress and subsequent heat strain on aerobic performance [130, 155, 165, 178] and muscle function [158, 164, 179], as well as mood states and perceived exertion [165, 180, 181], which may in turn impact aspects of team sport performance. It is important to note that the studies using heat and exercise to induce hypohydration also involved higher levels of BML. In addition, some of these studies included a rest period between the dehydrating exercise/heat protocol and the commencement of performance drills to allow body core temperature to return to baseline values [97, 102, 103]. Thus, it could be argued that hypohydration per se was responsible for the impaired performance. Nonetheless, in real life it can be difficult to separate out the effects of hypohydration and heat stress, as the two are closely linked when training/competing in warm–hot environments (i.e., exercise in the heat increases sweat rate thereby magnifying fluid losses) [9].

Another factor related to the method of dehydration is the timing of body water loss with respect to completion of the sport-specific protocol and performance tests. Most studies were designed to determine the effects of dehydration, accrued throughout sport-specific training/play, on performance. Mixed results were reported with this methodological approach. By contrast, some studies established hypohydration in the hours before [97, 102, 103] or in some cases the day before [98, 114] the sport-specific tests. A detrimental effect of hypohydration was reported in all five of these studies (albeit most of these studies also involved higher levels of hypohydration (>2% BML) and/or heat stress). This methodological approach allows for a more systematic investigation of the effects of hypohydration (e.g., a standard level of BML) and provides insight on performance effects when athletes begin training/competition in a hypohydrated state (which may be applicable for tournaments or back-to-back training sessions). However, it is not applicable to scenarios where athletes begin exercise in a euhydrated state, and, as such, impacts on the ecological validity of the study findings. It would be interesting for future research to directly compare the effects of previous dehydration versus in-game dehydration on performance.

The level of BML reached in hypohydration trials and the inclusion of a proper euhydration control trial are other important factors to consider. In the studies reviewed, most included a control (fluid intake) trial to compare performance against that of hypohydration (i.e., fluid restriction) trials. However, across studies, varying degrees of BML accrued during the control trials. That is, some studies aimed to replace fluid losses and maintain euhydration (< 1% BML [9]), while others involved ad libitum intake or prescribed a fixed volume of fluid intake that resulted in mild to moderate hypohydration in the control trials. For example, several studies in soccer dehydrated athletes to ~2–3% BML in the fluid restriction trials, but also accrued 1–2% BML in the fluid intake trials [95, 105, 106, 113]. By contrast, in most basketball [97, 102, 103], baseball [85, 114], and cricket [83, 109] studies, subjects maintained euhydration (< 1% BML) in the control trials. The differences in study design are likely due, in part, to attempts to implement ecologically valid fluid intake patterns, which reflect differences in drinking opportunities across sports. Nonetheless, the inconsistency makes it difficult to assimilate results across the literature.

For the purpose of this discussion (and Table 4), BML differences between control trials and hypohydration trials were calculated for each study. In this regard, most investigations involved a 1–2% BML difference between control trials and hypohydration trials. In these studies, there were mixed results in how hypohydration impacted cognition, sprinting, lateral movements, and intermittent running capacity, and no or little effect of hypohydration on jumping/power and skill. Only five studies involved a 3–4% BML difference between trials, but all found impaired performance with hypohydration [83, 85, 97, 102, 114]. It is important to note that these studies also involved exercise in the heat as the method of dehydration, so it is unclear whether the heat stress also contributed to the performance impairment. Nonetheless, two out of three studies involving graded levels of hypohydration (~ 1–4% BML) have indicated that ~3–4% BML was more likely to impair performance than ~1–2% BML [85, 97, 102].

**Fig. 2:**
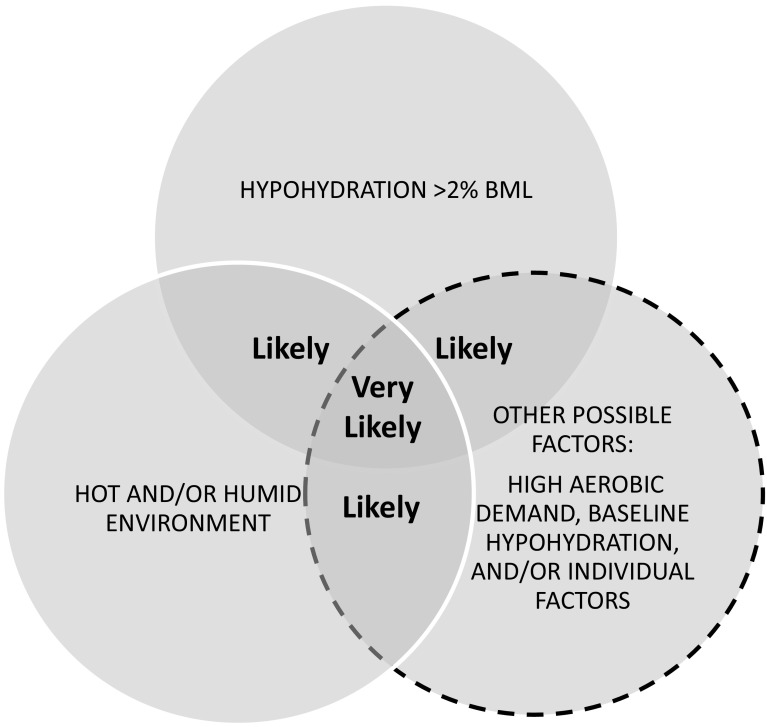
Venn diagram showing the likelihood of performance impairment with hypohydration. Based on the studies reviewed, the likelihood of performance impairment seems to increase with higher levels of hypohydration and heat stress. The* circle* with the* dashed line* represents other factors that may play a role, but require more research in team sports; these include high aerobic demand, hypohydration at baseline, and individual differences in the response to hypohydration (e.g., low cognitive resiliency). *BML* body mass loss

Figure 2 shows a Venn diagram illustrating the likelihood of team sport performance impairments with hypohydration. Based on the studies reviewed, decrements in cognitive, technical, or physical performance seem more likely with higher levels of hypohydration and heat stress. However, as discussed in Sects. 5 and 6, other factors may play a role, but currently lack sufficient research in team sports. These include high aerobic demand, hypohydration at baseline, and individual differences in the response to hypohydration (e.g., low cognitive resiliency).

## Study Limitations

The potential limitations of individual studies reviewed in this paper are described in Tables 2 and 3. One of the most common potential limitations is the inherent difficulty in blinding subjects to the fact that they are dehydrating (e.g., no fluid or strict fluid restriction) versus rehydrating (e.g., prescribed or ad libitum fluid intake) during a given trial. Because the awareness of being dehydrated may confound performance results, attempts should be made to disguise experimental conditions. For example, small volumes of fluid should be provided during dehydration trials and the subjects’ body mass, fluid intake, and urine volumes should be concealed [182]. Ganio et al. [134] employed similar techniques in a study investigating the effects of mild hypohydration without hyperthermia in men and found that 1.6% BML decreased vigilance and working memory and increased tension/anxiety and fatigue. While these masking techniques can be helpful, it remains difficult to effectively blind subjects to experimental conditions when attempting to induce higher levels of hypohydration (e.g., 3–4%).

Another common study limitation is related to the type of test used to measure the effect of hypohydration on performance. Many different tests have been used and, in some studies, limited information about the test was reported. Protocols and tests used to measure performance in team sports should be sport-specific (i.e., mimic the actual physical demands and skills required of the sport) and subjects should be familiarized with the methods prior to the start of experimental trials. The tests should also be valid, reliable, and sensitive [183]. Of the studies reviewed, most tests were sport-specific for skill, sprinting, jumping/power, lateral movements, and intermittent running capacity, but not for cognition (with the exception of one field hockey study [98]). Most cognitive tests were, however, valid and reliable standardized tests. The validity and reliability of skill tests were not reported in the basketball, tennis, and cricket studies reviewed, but were reported in the soccer and field hockey studies. Interestingly, the effect of hypohydration on skill performance was mixed whether validity and reliability were reported or not (see Table 2).

## Considerations for Future Directions

From the discussion above it is clear that more research is needed to address several remaining questions regarding the potential impact of hypohydration on team sport performance. First, valid, reliable, and sensitive sport-specific protocols should be developed and used in future studies to ensure that tests are able to detect small but meaningful differences in performance. In general, valid/reliable sport-specific tests to measure cognition and skill are currently limited in most sports.

Most studies have tested the effect of low–moderate levels of hypohydration on performance. In future studies, it would be helpful to include higher levels of hypohydration, perhaps in a graded manner. In addition, studies directly comparing the effect of hypohydration on different cohorts, such as male versus female, youth versus adults, or low- versus high-caliber athletes, would be helpful in determining who may be more susceptible to the detrimental effects of hypohydration, from both a physiological (heat safety) and a performance perspective. In most studies of the current literature the amount and pattern of fluid intake is controlled. However, in real life athletes often drink ad libitum. Thus, more studies should include an ad libitum fluid intake trial to compare against the effects of no fluid and prescribed intake to better understand the scenarios in which ad libitum may be sufficient versus when prescribed intake is warranted to maintain performance.

For all of the aforementioned research questions, it is particularly important that future studies focus on the sports that are associated with a moderate or high risk of developing significant hypohydration. Some examples of these sports include soccer, rugby, American Football, Australian Rules Football, field hockey, ice hockey, and tennis. By contrast, sports in which sweating rates are expected to be low and/or fluid replacement opportunities are adequate (e.g., baseball) probably warrant less investigation. Still, there may be certain players in low risk sports that have increased risk of developing hypohydration due to equipment requirements and/or the physical demands of the position (e.g., baseball catcher).

## Conclusion

Significant hypohydration (>2%) has been reported most consistently in soccer. Although other sports (e.g., American Football, rugby, basketball, tennis, and ice hockey) have reported high sweating rates, fluid balance disturbances have generally been mild, suggesting that drinking opportunities were sufficient to provide most athletes with enough fluid to offset losses. The effect of hydration status on team sport performance has been mixed. However, it seems that hypohydration is more likely to impair cognition, technical skill, and physical performance at higher levels of BML (3–4% difference between trials), which are not routinely observed in team sport athletes. Detriments to performance are also more likely when the method of dehydration involves heat stress. Although exact mechanisms are unclear, increased subjective ratings of fatigue and perceived exertion consistently accompany hypohydration in team sport studies and could explain, in part, the performance impairments reported in some studies. More research is needed to develop ecologically valid, reliable, and sensitive sport-specific protocols and should be used in future studies to determine the effects of hypohydration and modifying factors (e.g., age, sex, athlete caliber) on team sport performance.
